# Study on the relationship between disease progression and changes in Th17/Treg levels in patients with relapsing–remitting multiple sclerosis

**DOI:** 10.3389/fneur.2026.1887969

**Published:** 2026-08-07

**Authors:** Jinshui Yang, Qian Xue, Xiaoning Wang, Aixia Song

**Affiliations:** Department of Neurology, The First Affiliated Hospital of Hebei North University, Zhangjiakou, Hebei, China

**Keywords:** disease progression, immunomodulation, relapsing–remitting multiple sclerosis, Th17 cells, Treg cells

## Abstract

**Objective:**

This study aims to investigate the relationship between disease progression and changes in Th17/Treg levels in patients with relapsing–remitting multiple sclerosis (RRMS).

**Methods:**

This retrospective study analyzed clinical data and Th17/Treg cell levels from 130 RRMS patients and 100 non-inflammatory neurological disease controls treated between December 2021 and January 2025. Th17 cells were identified as CD4^+^IL-17A^+^ cells and Treg cells as CD4^+^CD25^+^Foxp3^+^ cells via flow cytometry.

**Results:**

The two groups were comparable in baseline characteristics (*p* > 0.05). The proportion of CD4^+^IL-17A^+^ Th17 cells was significantly higher in RRMS patients than in controls (4.26 ± 1.35% vs. 1.52 ± 0.47%, *p* < 0.05), while CD4^+^CD25^+^Foxp3^+^ Treg cells were significantly lower (2.91 ± 0.98% vs. 5.38 ± 1.52%, *p* < 0.05). Acute-phase patients showed higher Th17 and lower Treg proportions than remission-phase patients (*p* < 0.05). Following methylprednisolone treatment, Th17 cells decreased, Treg cells increased, and EDSS scores improved (*p* < 0.05 for all). Pearson correlation analysis revealed that Th17 proportion and Th17/Treg ratio were positively correlated with EDSS scores (*r* = 0.521, *p* < 0.01; *r* = 0.485, *p* < 0.001), while Treg proportion was negatively correlated (*r* = −0.387, *p* < 0.05).

**Conclusion:**

Th17/Treg imbalance may be an important mechanism underlying RRMS pathogenesis and disease progression. Modulating this balance may represent a promising therapeutic target for RRMS.

## 1 Introduction

Multiple Sclerosis (MS) is a chronic inflammatory demyelinating disease of the central nervous system, with Relapsing–Remitting Multiple Sclerosis (RRMS) being the most common clinical type, characterized by recurrent episodes of neurological dysfunction and remission periods (1, 2). The pathogenesis of RRMS is complex, involving abnormal immune system attacks on the myelin sheath of the central nervous system, leading to impaired neural signal transmission (3–6). Currently, it is believed that helper T cell 17 (Th17) and regulatory T cells (Treg) play important roles in the pathogenesis and disease progression of RRMS. Th17 cells are a subset of CD4 + T cells that exert pro-inflammatory effects in autoimmune diseases by secreting pro-inflammatory cytokines such as interleukin-17 (IL-17). Studies have shown that the proportion of Th17 cells in the peripheral blood of RRMS patients is elevated, and the level of IL-17 secreted by these cells is correlated with disease activity. Treg cells, on the other hand, are a subset of CD4 + T cells with immunoregulatory functions that maintain immune tolerance and prevent the occurrence of autoimmune diseases by inhibiting the activation of autoimmune T cells. In RRMS patients, the proportion of Treg cells is decreased, and their function may also be impaired.

Th17 and Treg cells are antagonistic to each other in terms of differentiation and function, jointly maintaining immune homeostasis. Th17/Treg imbalance may lead to abnormal activation of the immune system and trigger autoimmune diseases (7). In RRMS, hyperfunction of Th17 cells and hypofunction ofTreg cells may be associated with disease onset and progression. However, the specific relationship between Th17/Treg imbalance and RRMS disease progression remains to be further investigated. This study retrospectively analyzed the clinical data and changes in Th17/Treg cell levels in RRMS patients and patients with non-inflammatory neurological diseases during the same period, aiming to explore the relationship between Th17/Treg imbalance and RRMS disease progression and provide new insights into the pathogenesis and immunotherapy of RRMS.

## 2 Object and method

### 2.1 Research object

This study selected RRMS patients who were treated in our hospital between December 2021 and January 2025 as the research subjects to ensure sample representativeness. A total of 130 RRMS patients meeting the predefined inclusion and exclusion criteria were identified from the hospital’s medical record system and included in the experimental group. Among the 130 RRMS patients, 78 were in the acute phase and 52 were in the remission phase at the time of initial blood sampling. Concurrently, 100 patients with non-inflammatory neurological diseases who were hospitalized in the Neurology Department of our hospital during the same period were selected as the control group. These control patients were also identified from medical records and frequency-matched to the experimental group by age and sex. The specific diagnoses of the control group included: migraine (*n* = 28), tension-type headache (*n* = 19), epilepsy (*n* = 15), peripheral neuropathy (*n* = 12), vestibular neuritis (*n* = 10), carpal tunnel syndrome (*n* = 8), and benign paroxysmal positional vertigo (*n* = 8) —all diagnosed according to established international diagnostic criteria (ICHD-3 for headaches, ILAE for epilepsy, etc.) as documented in the medical records. This was designed as a retrospective study. The use of de-identified clinical data and stored biospecimens for research purposes was approved by the Ethics Committee of our hospital, and the study adhered to the Declaration of Helsinki. Given that this was a retrospective study utilizing existing clinical data, the Ethics Committee of our hospital waived the requirement for informed patient consent.

### 2.2 Inclusion criteria

Inclusion criteria: Recent presentation of positive signs or new clinical symptoms; detection of new enhancing lesions by cranial magnetic resonance imaging (MRI); adult age range (≥18 years); availability of complete clinical medical records and peripheral blood samples.

Exclusion criteria included: Patients with concomitant autoimmune diseases; confirmed infectious diseases or active malignant tumors; patients who received immunosuppressants or glucocorticoid therapy within the past 3 months; patients with hypertension, diabetes, active peptic ulcer, chronic liver disease, or liver dysfunction. Patients whose medical records lacked key clinical or laboratory data required for analysis were also excluded.

### 2.3 Treatment methods

Control group protocol: Patients received conventional supportive medical measures after admission, with standardized symptomatic treatment based on clinical symptoms. “Conventional supportive medical measures” included analgesics (for headache management), vitamins (B-complex vitamins), and symptomatic treatment for the underlying neurological conditions. None of these medications are known to significantly modulate Th17 or Treg cell populations.

Experimental group intervention protocol: All RRMS patients in the acute phase received methylprednisolone intravenous intensive therapy according to the standard institutional protocol, as documented in their medical records. The regimen was: 1000 mg/d for 72 h; Days 4–6: Dose adjusted to 500 mg/d; subsequently, the dose was reduced by 50% every 72 h, decreasing sequentially to 250 mg/d → 125 mg/d → 62.5 mg/d. When the intravenous dose was reduced to 30 mg/d, it was switched to oral prednisone therapy, with an initial maintenance dose of 35 mg/d; the prednisone dose was reduced by 5 mg weekly until final discontinuation.

### 2.4 Detection methods

All peripheral blood samples analyzed in this study were residual specimens originally collected as part of routine clinical management of patients at our center. Specifically, for the experimental group (RRMS patients), blood samples were drawn at two standardized time points that correspond to our hospital’s established clinical protocol for managing acute relapses: (1) within 24 h prior to initiation of methylprednisolone pulse therapy (pre-treatment baseline), and (2) upon completion of the intravenous steroid course (day 5–7 post-initiation), which coincides with the timing for routine clinical reassessment and laboratory monitoring of patients receiving high-dose corticosteroid therapy. For control subjects, blood samples were collected at the time of hospital admission as part of their standard diagnostic workup. Following completion of all clinically indicated tests, any remaining blood specimens were stored at −80 °C in our institutional biobank with prior patient consent for future research use.

For this retrospective study, de-identified aliquots were retrieved from the biobank and processed according to the following protocol. A total of 3 mL of whole blood was used for flow cytometry analysis. Peripheral blood mononuclear cells (PBMCs) were isolated using density gradient centrifugation with Ficoll-Paque PLUS (GE Healthcare) after mixing blood samples with an equal volume of PBS buffer in BD centrifuge tubes. Post-thaw cell viability was assessed by trypan blue dye exclusion assay using a hemocytometer; only samples with viability ≥70% were included in the subsequent flow cytometric analysis (8). For Th17 cell detection, PBMCs were stimulated with 50 ng/mL phorbol 12-myristate 13-acetate (PMA) and 500 ng/mL ionomycin in the presence of 10 μg/L brefeldin A for 4 h at 37 °C in a 5% CO₂ incubator. For Treg cell detection, PBMCs were analyzed without stimulation.

Flow cytometric acquisition was performed on a BD FACSCanto II flow cytometer (BD Biosciences). The instrument was calibrated daily prior to sample testing using BD Cytometer Setup and Tracking (CST) beads. For each specimen, a minimum of 100,000 events within the lymphocyte gate were collected. The sequential gating strategy was as follows: (1) cellular debris and dead cells were excluded by gating lymphocytes according to forward scatter area (FSC-A) versus side scatter area (SSC-A); (2) doublets were excluded using FSC-A versus FSC-width (FSC-W) dot plots; (3) CD3^+^ T lymphocytes were identified based on CD3-APC-Cy7 (clone SK7, BD Biosciences) expression; (4) CD4^+^ T helper cells were further gated from the CD3^+^ T cell population; (5) Th17 cells were defined as CD3^+^CD4^+^IL-17A^+^ cells within the CD3^+^CD4^+^ gate; (6) Treg cells were defined as CD3^+^CD4^+^CD25^+^Foxp3^+^ cells within the CD3^+^CD4^+^ gate. All reported frequencies of Th17 and Treg cells are presented as proportions of CD3^+^CD4^+^ T lymphocytes. Isotype-matched control antibodies at equivalent concentrations were used to evaluate non-specific antibody binding. In addition to isotype controls, fluorescence-minus-one (FMO) controls were prepared for each fluorescence channel. Cells were stained with the full antibody panel omitting one target antibody each time, followed by identical fixation and permeabilization procedures as experimental samples. FMO controls were applied to define precise positive/negative cutoffs for IL-17A-PE and Foxp3-FITC, given the relatively low expression of these markers and their susceptibility to fluorescence spillover in multi-color flow cytometry panels. Representative sequential gating plots are displayed in Supplementary Figure S1. BD FACSDiva software (version 8.0, BD Biosciences) was utilized to calculate Th17 and Treg cell percentages. All PBMC samples from RRMS patients and controls were cryopreserved at −80 °C, thawed and processed using identical protocols. Therefore, any potential influences of long-term cryopreservation on cell viability or T-cell subset composition would exert systematic and non-differential effects between the two groups (9).

### 2.5 Data analysis

The images in this study were processed using GraphPad Prism 8. Data were organized and analyzed using SPSS 26.0. Measurement data were expressed as mean ± standard deviation (±s), and the t-test was used for intergroup comparisons; counting data were expressed as [*n* (%)], and the *χ*^2^ test was used for intergroup comparisons. Paired *t*-tests were used for pre versus post-treatment comparisons within the experimental group. Pearson correlation analysis was used to investigate the correlation between Th17/Treg levels and disease severity as measured by EDSS scores. EDSS scores were extracted from the patients’ medical records, where they had been assessed by attending neurologists at the time of admission (pre-treatment) and at follow-up visits (post-treatment). A *p* < 0.05 was considered statistically significant. Missing data points were handled by pairwise exclusion, and the number of available cases for each analysis is specified in the respective results sections.

## 3 Results

### 3.1 General characteristics

A total of 130 RRMS patients meeting the predefined inclusion and exclusion criteria were identified from the hospital’s electronic medical record system and included in the experimental group. Among them, 42 were males and 88 were females, aged 20–55 years (mean age: 35.28 ± 8.61 years). Education levels: 26 high school, 39 college, and 65 bachelor’s degree. The control group consisted of 100 patients with non-inflammatory neurological diseases, also identified from the medical records during the same period (35 males and 65 females, aged 22–58 years, mean age, 36.59 ± 9.17 years). Education levels: 11 high school, 31 college, and 58 bachelor’s degree. Among the experimental group, 78 patients (60.0%) were documented as being in the acute phase and 52 patients (40.0%) in the remission phase at the time of initial blood sampling, as recorded in the medical charts. The two groups were comparable in gender, age, and other general characteristics (*p* > 0.05). All demographic and clinical data presented in this section were extracted from the hospital’s electronic medical record system. See Table 1.

**Table 1:** Comparison of general characteristics between the two groups.

Characteristic subcategory		Experimental group	Control group	*t*	*P*
Number of Cases		130	100	—	—
Gender	Male	42	35	—	—
	Female	88	65	—	—
Age (years)	Range	20–55	22–58	—	—
	Mean	35.28 ± 8.61	36.59 ± 9.17	1.112	0.267
Education levels	High school	26	11	—	—
	Associate degree	39	31	—	—
	Bachelor’s degree	65	58	—	—
Disease phase (experimental)	Acute	78	—	—	—
	Remission	52	—	—	—

### 3.2 Changes in Th17 and Treg cell proportions

The proportions of CD4^+^IL-17A^+^ Th17 cells and CD4^+^CD25^+^Foxp3^+^ Treg cells in peripheral blood derived from the retrospectively retrieved samples, as well as the Th17/Treg ratio, were compared between the experimental and control groups, and between acute-phase and remission-phase patients within the experimental group. All comparisons were statistically significant (*p* < 0.05). See Table 2.

**Table 2:** Comparison of Th17 and Treg cell proportions between groups and across disease phases.

Group	*n*	CD4^+^IL-17A^+^ Th17 cells (% of CD4^+^ T cells)	CD4^+^CD25^+^Foxp3^+^ Treg cells (% of CD4^+^ T cells)	Th17/Treg ratio
Control group	100	1.52 ± 0.47	5.38 ± 1.52	0.29 ± 0.11
Experimental group (total)	130	4.26 ± 1.35*	2.91 ± 0.98*	1.53 ± 0.62*
Acute phase	78	5.18 ± 1.21#	2.21 ± 0.76#	2.41 ± 0.87#
Remission phase	52	2.87 ± 0.92	3.96 ± 1.04	0.75 ± 0.31

Data are presented as mean ± standard deviation. * *P* < 0.05 vs. control group; # *p* < 0.05 vs. remission phase.

### 3.3 Treatment outcomes

#### 3.3.1 EDSS scores

EDSS scores were extracted from the patients’ medical records, where they had been assessed by attending neurologists at the time of admission (pre-treatment) and at follow-up visits conducted at 4 weeks following treatment initiation (post-treatment). The mean EDSS score decreased significantly after methylprednisolone treatment (*p* < 0.05). See Table 3.

**Table 3:** Changes in Th17/Treg cell proportions and EDSS scores before and after methylprednisolone treatment in RRMS patients (*n* = 130).

Time point	CD4^+^IL-17A^+^ Th17 cells (%)	CD4^+^CD25^+^Foxp3^+^ Treg cells (%)	Th17/Treg ratio	EDSS score
Pre-treatment	5.18 ± 1.21	2.21 ± 0.76	2.41 ± 0.87	3.8 ± 1.2
Post-treatment	2.65 ± 0.88*	4.12 ± 1.13*	0.66 ± 0.28*	2.4 ± 1.1*

Data are presented as mean ± standard deviation. * *p* < 0.05 vs. pre-treatment (paired *t*-test).

#### 3.3.2 Changes in Th17 and Treg cell proportions after treatment

Based on flow cytometric analysis of the retrieved peripheral blood samples, the proportion of CD4^+^IL-17A^+^ Th17 cells decreased significantly after treatment, while the proportion of CD4^+^CD25^+^Foxp3^+^ Treg cells increased significantly. Consequently, the Th17/Treg ratio decreased significantly after treatment (*p* < 0.05 for all comparisons), consistent with the clinical improvement documented in the medical records. See Table 3.

### 3.4 Correlation analysis

Pearson correlation analysis based on the retrospectively extracted clinical and laboratory data revealed that the proportion of CD4^+^IL-17A^+^ Th17 cells and the Th17/Treg ratio in the experimental group were positively correlated with EDSS scores, while the proportion of CD4^+^CD25^+^Foxp3^+^ Treg cells was negatively correlated with EDSS scores. See Table 4.

**Table 4:** Correlation between Th17/Treg cell proportions and disease severity (EDSS scores).

Cell population	*r*	*P*
CD4^+^IL-17A^+^ Th17 cells (% of CD4^+^ T cells)	0.521	<0.01
CD4^+^CD25^+^Foxp3^+^ Treg cells (% of CD4^+^ T cells)	−0.387	<0.05
Th17/Treg ratio	0.485	<0.001

## 4 Discussion

Based on existing clinical research findings, when naive CD4^+^ T lymphocytes encounter exogenous antigenic stimulation, they initiate proliferation and differentiation programs to form multiple effector T cell subsets. Among these, the synergistic action of IL-12 and IFN-*γ* drives differentiation toward the Th1 lineage, which secretes IFN-γ to enhance cellular immune responses and plays a critical role in anti-infection and antitumor immune surveillance. Conversely, IL-4 induces polarization toward the Th2 phenotype, with these cells participating in humoral immune regulation through the release of cytokines such as IL-4, IL-5, and IL-13, while also being closely associated with the pathogenesis of hypersensitivity reactions and systemic autoimmune diseases. TGF-*β* alone promotes the differentiation of naive T cells into regulatory T cells (Treg), which maintain immune homeostasis by autocrine secretion of TGF-*β* and expression of the Foxp3 transcription factor (10). Notably, recent studies have identified Th17 cells as a novel effector subset characterized by IL-17 secretion, which is deeply involved in the regulatory network of inflammatory responses (11, 12). It is worth emphasizing that dynamic equilibrium exists between Treg cell-mediated immune tolerance and Th17 cell-induced inflammatory responses. Under physiological conditions, this equilibrium is finely balanced to ensure immune homeostasis. However, when functional dysregulation occurs, this balance is often disrupted, manifesting as a Treg/Th17 ratio imbalance that triggers inflammatory cascades leading to tissue damage (13). In the present retrospective study, we analyzed peripheral blood samples from 130 RRMS patients and 100 non-inflammatory neurological disease controls to investigate the relationship between Th17/Treg imbalance and disease progression.

The results of this study show that the proportion of CD4^+^IL-17A^+^ Th17 cells in peripheral blood was significantly higher in the experimental group than in the control group, with acute-phase patients exhibiting significantly higher Th17 cell proportions than remission-phase patients. This suggests that Th17 cell hyperfunction may be an important mechanism in the pathogenesis and progression of RRMS. By secreting pro-inflammatory cytokines such as IL-17, Th17 cells play a significant role in the onset and progression of RRMS. IL-17 induces the activation of other inflammatory cells, promotes the release of inflammatory factors, and exacerbates inflammatory damage in the central nervous system, as previously demonstrated in both experimental autoimmune encephalomyelitis and MS patients (14, 15). Further analysis revealed a positive correlation between CD4^+^IL-17A^+^ Th17 cell percentage and EDSS scores in the experimental group, indicating that higher Th17 cell proportions correlate with more severe disease. Post-treatment, the proportion of CD4^+^IL-17A^+^ Th17 cells decreased in the experimental group, accompanied by clinical symptom improvement, further supporting the role of Th17 cells in RRMS pathogenesis and progression.

CD4^+^CD25^+^Foxp3^+^ Treg cells maintain immune tolerance and prevent autoimmune diseases by inhibiting the activation of autoimmune T cells (16). In RRMS patients, reduced Treg cell proportions and impaired function may lead to an imbalance between immunosuppression and pro-inflammatory responses (17). The results of this study show that the proportion of CD4^+^CD25^+^Foxp3^+^ Treg cells in peripheral blood was significantly lower in the experimental group than in the control group, with acute-phase patients exhibiting significantly lower Treg cell proportions than remission-phase patients. This suggests that Treg cell dysfunction may be another important mechanism in RRMS pathogenesis and progression. Further analysis revealed a negative correlation between CD4^+^CD25^+^Foxp3^+^ Treg cell percentage and EDSS scores in the experimental group, indicating that lower Treg cell proportions correlate with more severe disease. Post-treatment, CD4^+^CD25^+^Foxp3^+^ Treg cell proportions increased in the experimental group, accompanied by clinical symptom improvement, further supporting the role of Treg cells in RRMS pathogenesis and progression.

Previous scholars have proposed that Th17 and Treg cells are antagonistic in differentiation and function, jointly maintaining immune homeostasis. Th17/Treg imbalance may lead to abnormal immune activation and autoimmune diseases (18). In RRMS, CD4^+^IL-17A^+^ Th17 cell hyperfunction and CD4^+^CD25^+^Foxp3^+^ Treg cell dysfunction may increase the release of inflammatory factors, promote inflammatory cell infiltration into the central nervous system, and aggravate nerve myelin damage, thereby accelerating disease progression. The results of this study show that the Th17/Treg ratio (CD4^+^IL-17A^+^/CD4^+^CD25^+^Foxp3^+^) was significantly higher in the experimental group than in the control group, with a positive correlation between the Th17/Treg ratio and EDSS scores, suggesting that Th17/Treg imbalance may be an important mechanism in RRMS pathogenesis and progression. Post-treatment, the Th17/Treg ratio decreased in the experimental group, accompanied by clinical symptom improvement, further supporting this hypothesis.

Regarding the effects of methylprednisolone on Th17 and Treg cells, the literature presents a complex and sometimes contradictory picture that warrants careful examination. On one hand, several studies have reported immunomodulatory effects consistent with our observations. Braitch et al. (19) demonstrated that in MS patients during relapse, the percentage of CD4^+^CD25^+^ cells, plasma IL-10 levels, and the Foxp3/CD3 ratio all increased significantly at 48 h after methylprednisolone initiation, suggesting that glucocorticoids enhance Treg cell functional molecules and percentages—a mechanism that may expedite recovery from MS relapses. Similarly, Hecker et al. (20) reported that high-dose intravenous methylprednisolone treatment in MS patients during relapse induced significant transcriptomic changes in CD4^+^ T helper cells, with 55 genes significantly upregulated or downregulated following therapy. In experimental autoimmune encephalomyelitis (EAE) models, methylprednisolone has been shown to regulate the gene expression of CD4^+^ T lymphocytes, induce Treg cell proliferation, and reduce the secretion of pro-inflammatory cytokines (21). Furthermore, methylprednisolone has been reported to inhibit the entry of T cells into the central nervous system, suppress pro-inflammatory T cell activity, and promote Treg cell activity. Wu et al. (22) found that MP treatment increased the number of Tfr cells (a subset of Treg cells) and reduced Tfh cells, thereby regulating the imbalanced Tfr/Tfh ratio in EAE mice through inhibition of the PI3K/AKT/FoxO1 and PI3K/AKT/mTOR signalling pathways.

On the other hand, studies have reported findings that appear to contradict or complicate this picture. Muls et al. (23) observed that while intravenous methylprednisolone decreased the overall proportion of Tregs in MS patients, it concurrently increased CD39 mRNA levels, the proportions of CD39-expressing Tregs and monocytes, as well as CD39 ectonucleotidase activity. This suggests that the effect of corticosteroids on Tregs may involve qualitative changes in Treg function (e.g., enhanced CD39-mediated immunosuppressive capacity) rather than simply a quantitative increase in Treg numbers. Additionally, some studies have reported no significant increases in Treg cell percentages in relapsing MS patients after methylprednisolone pulse therapy. Research by Martínez et al. (24) demonstrated that while MP treatment induced immediate post-treatment effects on the immune system, these changes were not sustained at 6 months post-therapy, suggesting that the immunomodulatory effects of corticosteroids may be transient rather than durable.

These discrepant findings may reflect several important factors. First, the disease phase at the time of sampling—relapsing versus remitting—may influence the baseline immune profile and thus the response to corticosteroid therapy. Second, the timing of sample collection relative to treatment initiation appears critical: Braitch et al. (19) observed increased Treg percentages at 48 h, which returned to baseline by 6 weeks, whereas our study measured Tregs at day 5–7 post-treatment, which may capture a different phase of the immune response. Third, the specific Treg markers used for identification vary across studies—CD4^+^CD25^+^, CD4^+^CD25^+^Foxp3^+^, and CD4^+^CD25^+^CD127^−^Foxp3^+^—and may identify overlapping but not identical cell populations with potentially different functional properties (25). Fourth, individual patient variability in treatment response, as well as differences in corticosteroid dosing regimens and routes of administration, may contribute to the observed heterogeneity.

Our observation that methylprednisolone treatment was associated with decreased CD4^+^IL-17A^+^ Th17 cells and increased CD4^+^CD25^+^Foxp3^+^ Treg cells is consistent with the immunomodulatory effects reported in several of the above studies. However, we acknowledge that the effect on Tregs may be more nuanced than a simple numerical increase. The present study measured Tregs as CD4^+^CD25^+^Foxp3^+^ cells at a single post-treatment time point (day 5–7), which may not capture the full temporal dynamics of Treg modulation. Future studies incorporating multiple post-treatment sampling time points and functional assays of Treg suppressive capacity would help to further elucidate the complex relationship between corticosteroid therapy and Treg biology in RRMS.

Existing research indicates significant immune regulatory network imbalances in the peripheral blood microenvironment of multiple sclerosis (MS) patients (26). When the number and function of immunomodulatory CD4^+^IL-17A^+^ Th17 cells decline, while the Treg-suppressed Th17 subset compensatorily expands and becomes overactivated, this imbalance triggers pathophysiological changes. Specifically, overactivated Th17 cells secrete large amounts of pro-inflammatory cytokines such as IL-17, activating autoimmune cascades that directly attack the myelin sheath of the central nervous system, causing demyelination and axonal damage. The persistent progression of this neuroimmune damage ultimately leads to the characteristic neurological dysfunction and clinical manifestations of MS, consistent with the findings of this study.

This study has several limitations that should be acknowledged. First, as a retrospective clinical analysis, it is subject to inherent biases associated with the use of existing medical records and stored bio specimens. Second, the absence of a healthy control group is a notable limitation; while the non-inflammatory neurological disease controls are clinically useful for distinguishing MS-specific effects from general neurological illness, this design cannot fully define “normal” Th17/Treg reference ranges or completely exclude the influence of hospitalization, stress, or medications on peripheral immune profiles. Third, this study only examined CD4^+^IL-17A^+^ Th17 and CD4^+^CD25^+^Foxp3^+^ Treg cell proportions in peripheral blood, without assessing their levels or functional status in the central nervous system, which may not fully reflect disease status in the target organ. Fourth, the single post-treatment time point for immune measurements (day 5–7) may not capture the full temporal dynamics of immune modulation following corticosteroid therapy. Fifth, the relatively modest sample size, particularly in subgroup analyses (acute phase: *n* = 78; remission phase: *n* = 52), limits the statistical power for detecting smaller effect sizes. Future studies with larger prospective cohorts, inclusion of healthy controls, multiple post-treatment sampling time points, and assessment of immune cell function in the central nervous system would help to further validate and extend these findings. Sixth, the present study used isotype controls to establish positivity thresholds for Th17 and Treg cell identification. While isotype controls can provide information on non-specific antibody binding, they do not account for fluorescence spillover from other channels—a major source of background signal in multicolor flow cytometry. For low-frequency populations such as IL-17A^+^ cells and Foxp3^+^ Tregs, FMO controls are currently considered more appropriate for defining accurate positive/negative boundaries. Although FMO controls were prepared and used for gate placement in this study, the absence of a dedicated comparison between isotype-based and FMO-based gating approaches represents a methodological limitation. Future studies should consider reporting both approaches and relying primarily on FMO controls for threshold definition in multicolor panels analyzing rare T-cell subsets.

Despite these limitations, this study provides new insights into the pathogenesis and immunotherapy of RRMS. The consistent association between Th17/Treg imbalance and disease severity across multiple analytical approaches—cross-sectional comparison, phase-based subgroup analysis, and pre-post treatment comparison—strengthens the evidence for a potential role of this immune axis in RRMS. Modulating Th17/Treg balance may represent a promising avenue for therapeutic intervention in RRMS. Future research could explore immune intervention strategies targeting CD4^+^IL-17A^+^ Th17 and CD4^+^CD25^+^Foxp3^+^ Treg cells, such as using Th17 cell inhibitors or Treg cell agonists, to improve clinical outcomes in RRMS patients.

## 5 Conclusion

In summary, this retrospective study analyzed clinical data and CD4^+^IL-17A^+^ Th17 / CD4^+^CD25^+^Foxp3^+^ Treg cell level changes in 130 RRMS patients and concurrent non-inflammatory neurological disease controls. It was found that the experimental group exhibited elevated CD4^+^IL-17A^+^ Th17 cell proportions, reduced CD4^+^CD25^+^Foxp3^+^ Treg cell proportions, and a positive correlation between the Th17/Treg ratio and disease severity. Post-treatment, CD4^+^IL-17A^+^ Th17 cell proportions decreased, CD4^+^CD25^+^Foxp3^+^ Treg cell proportions increased, and the Th17/Treg ratio decreased in the experimental group, accompanied by clinical symptom improvement. These findings suggest that Th17/Treg imbalance may be an important mechanism in RRMS pathogenesis and progression, and modulating this balance warrants further investigation as a potential therapeutic strategy. Future prospective studies are needed to further validate these results and explore immune intervention strategies targeting CD4^+^IL-17A^+^ Th17 and CD4^+^CD25^+^Foxp3^+^ Treg cells to provide more effective treatment options for RRMS patients.

## Data Availability

The original contributions presented in the study are included in the article/Supplementary material, further inquiries can be directed to the corresponding author.

## Supplementary material

SUPPLEMENTARY FIGURE S1 Representative flow cytometry gating strategy for identification of Th17 and Treg cells. **(A)** Lymphocytes were first gated based on forward scatter area (FSC-A) versus side scatter area (SSC-A) to exclude cellular debris and dead cells. **(B)** Doublets were excluded using FSC-A versus FSC-width (FSC-W) dot plots to retain singlets. **(C)** CD3^+^ T lymphocytes were identified within the singlet population based on CD3-APC-Cy7 expression. **(D)** CD4^+^ T helper cells were further gated from the CD3^+^ T cell population. **(E)** Th17 cells were defined as CD3^+^CD4^+^IL-17A^+^ cells within the CD3^+^CD4^+^ T cell gate. **(F)** Treg cells were defined as CD3^+^CD4^+^CD25^+^Foxp3^+^ cells within the CD3^+^CD4^+^ T cell gate. Numerical values within each gate represent the percentage of the target population relative to the corresponding parent gate. Isotype controls together with fluorescence-minus-one (FMO) controls were applied to set positive/negative thresholds for each marker. Data are shown from a representative acute-phase RRMS patient sample.
